# Theoretical and Experimental Study of Energy-Harvesting and Movement-Sensing Solutions in Pneumatic Systems

**DOI:** 10.3390/s24237732

**Published:** 2024-12-03

**Authors:** Monica Tiboni, Federico Scassola, Alessandro Zanacchi, Marco Ghidini

**Affiliations:** 1 Department of Mechanical and Industrial Engineering, University of Brescia, Via Branze, 38, 25123 Brescia, Italy; federico.scassola@unibs.it; 2 Camozzi Automation s.p.a., via Eritrea, 20/I, 25126 Brescia, Italy; azanacchi@camozzi.com (A.Z.); mghidini@camozzi.com (M.G.)

**Keywords:** energy recovering, pneumatic actuators, end-stroke sensor, genetic algorithms, system modeling

## Abstract

This paper presents an experimentally based study aimed at assessing the viability of employing a commercial energy harvester to develop a self-powered end-stroke and speed sensor for pneumatic cylinders. An energy-harvesting device was integrated into a cylinder end-cap to recover energy from the piston impact at the end of the stroke. The recovered energy powers a radio transmitter that communicates the reach of the end-stroke. This avoids the use of a dedicated end-stroke sensor, reducing the number of components in the system and also saving energy. The experiments aimed to analyze the signal characteristics generated by the module at various activation speeds, assessing whether the impact speed could be distinguished from the signal. Energy output and short-term usage effects were also investigated. The study seeks to further develop and adapt a Simulink model of the system, based on recent studies, and validate it with experimental findings at the tested activation speeds. Following confirmation of the adapted model’s validity, the authors propose using genetic algorithms to design an optimized mechanical energy harvester. This approach aims to find the parameters of an energy harvester more suitable for pneumatic cylinder applications that would enable enhanced energy extraction and overall improved performances.

## 1. Introduction

Energy harvesting refers to the process of capturing and converting ambient or wasted energy from the surrounding environment into usable electrical power. Energy saving and recovery in industrial machinery are garnering growing interest in research, primarily due to their significant implications for environmental and energy sustainability. This applies to all drive technologies, including electrical [1,2], pneumatic [3,4,5,6], and hydraulic [7,8,9,10] systems.

In recent years, several studies have been dedicated to exploring different energy-harvesting solutions [11,12,13,14,15,16,17,18,19]. The primary focus of energy harvesting is the development of autonomous, miniaturized electronic circuits and sensing platforms that reduce or eliminate the need for battery replacement in low-power devices.

Energy-harvesting solutions are increasingly employed to power wireless sensor networks (WSNs) for condition-monitoring applications [20,21,22]. These systems provide a sustainable alternative to battery-powered sensors, addressing challenges like limited battery life and the need for frequent maintenance. These energy-harvesting techniques enable the deployment of autonomous and maintenance-free WSNs, significantly improving the feasibility and scalability of condition-monitoring systems in industrial applications.

Some devices utilize the complementary properties of different sources to harvest multiple types of ambient energy. In [23], a platform architecture, a reconfigurable multi-input, multi-output switching matrix combining energy from energy-harvesting sources (photovoltaics, thermoelectrics, and piezoelectrics) is presented. A thermoelectric and photovoltaic hybrid interface with an inductor is proposed by Liu et al. [11]. Huang, et al. [24] present a self-powered reconfigurable CMOS multisensor SoC for biomedical applications that integrates a dual-input power-harvesting interface with a conversion efficiency of 73% to harvest light energy and RF power.

In addition, the kinetic energy of mechanical or body movements can be captured and utilized. The three most important transducers used to capture kinetic energy are piezoelectric [25,26,27,28,29], electromagnetic [30,31], and electrostatic [26].

Piezoelectric, electromagnetic, and electrostatic energy harvesters differ in their working principles, efficiency, and suitable applications.

Piezoelectric harvesters generate electricity through the direct piezoelectric effect, where certain materials produce an electric charge in response to mechanical stress. They are compact, efficient for high-frequency vibrations, and require minimal external circuitry, making them ideal for applications like structural health monitoring and wearable devices. However, their power output is limited by the material properties.

Electromagnetic harvesters rely on Faraday’s law of electromagnetic induction, converting mechanical motion into electrical energy using a coil and a moving magnet. These devices are robust and can generate higher power levels, especially at lower frequencies, making them suitable for larger-scale applications like powering sensors in industrial machinery. However, they are bulkier and more complex than piezoelectric harvesters.

Electrostatic harvesters exploit the relative motion between charged surfaces to generate energy through changes in capacitance. They are lightweight and scalable, suitable for low-frequency vibrations and micro-scale applications, such as powering MEMS (Micro-Electro-Mechanical Systems). However, they typically require an external voltage source to pre-charge the surfaces, adding complexity to the system.

Each technology has distinct advantages and limitations, with the choice depending on the specific application requirements, such as size, frequency range, and power demand.

An overview of electromagnetic and piezoelectric harvesters of kinetic energy is presented in [32].

In the context of pneumatic systems, energy-harvesting technologies stand as viable solutions aimed at recuperating the otherwise lost energy from compressed air losses and impacts within pneumatic cylinders. The recovered energy can enable energy savings, as it can be used to operate devices that would otherwise require an external power supply. For example, the harnessed energy can power self-sufficient sensors and eliminates the need for external power sources. This offers a significant advantage for devices situated in remote or challenging-to-access locations where electricity connection is impractical or expensive in terms of cost and space. Additionally, it offers a compelling alternative to batteries, which might be arduous to replace. Consequently, these technologies enable wireless, self-powered sensors, allowing seamless device communication while reducing maintenance and enhancing long-term reliability [33]. Moreover, by reducing energy wastage and utilizing recovered energy effectively, energy-harvesting technologies pave the way for sustainable and eco-friendly practices [34].

Some of the studies on energy harvesting focus specifically on solutions tailored to the pneumatic sector, while others have a broader range of applications but are also promising in this context. These technologies exploit the different operating principles described above. Wang et al., for example, have developed a piezoelectric energy harvester that generates energy from the changing air pressure in a pneumatic system [35]. The device consists of an energy chamber, a pressure chamber, and a piezoelectric patch attached to the bottom of the energy chamber. It generates energy by deforming the piezoelectric patch. A similar solution integrating insulating elements was proposed by Yang et al. [36]. To explore other technologies, Cai et al. proposed a triboelectric nanogenerator in the form of a U-shaped tube made of fluorinated ethylene-propylene and copper foil to directly harvest energy from unused compressed air [37]. Esch et al. developed a foil-based, inductive energy collector that is attached to the outside of the pneumatic cylinder and utilizes the induced voltage generated by the movement of the magnet integrated in the piston [38].

Yang et al. [36] present a novel piezoelectric energy harvester that can be connected to the branch pipe to capture the air pressure impact energy in the pneumatic system.

Shveda et al. [39] have developed a textile-based energy-harvesting system to generate and store the energy needed to operate pneumatic actuators of assistive and rehabilitation devices. The energy is generated during walking via a soft textile pump that is integrated directly into the insole of the user’s shoe.

Another interesting technology is based on quasi-static mechanical energy harvesters (QST-MEH). These devices are designed for application in a wide application of self-powered transmitters. They have a switch which, when pressed, generates a considerable amount of electrical energy through its inductance, even with slow switching movements. The advantages of this technology are its simplicity, very low cost, relatively high output voltage, and frequency independence. Liu et al. in [40] recently conducted studies to understand the operational dynamics of these devices, which led to the development of a Simulink model capable of simulating the waveforms generated by QST-MEH devices during quasi-static activations.

As part of this research, one of the main objectives is to assess the feasibility of developing a self-powered end-stroke sensor using an electromagnetic energy-harvesting module. This solution could enable the conversion of the energy generated by the piston impact into an electrical signal, making the sensor entirely self-sufficient in terms of energy and eliminating the need for an external power source. This innovation would represent a significant step forward toward more efficient and sustainable monitoring and control systems, particularly relevant for industrial applications characterized by challenging operating conditions. In this context, a solution is presented in which a commercially available QST-MEH, the ECO 200 from EnOcean in conjunction with the PTM 535 module, is integrated into the end cap of a pneumatic cylinder. When the piston hits the end cap, it activates the ECO 200 module; the produced energy is used to power the Bluetooth radio transmitter of the PTM 535 module that can signal the reach of the end stroke.

The experiments described in this article also aimed to further investigate the feasibility of an energy-harvesting system based on a QST-MEH device for developing a self-powered velocity sensing system in a pneumatic application. The energy harvester would generate a signal whose characteristics depend on the impact velocity of the piston. This signal could potentially be processed by dedicated electronics (to be developed in future studies) to determine the actual impact velocity and transmit it to a receiver.

To ensure this possibility, it is crucial that the activation speed can be recognized from the signals generated by the QST-MEH. It is equally important to understand the energy output at different activation velocities.

The experiments measured the signals generated by the ECO 200 module when it was activated by a pneumatic cylinder at different speeds. Several waveforms were extrapolated to check if there is a correlation between them and the impact velocity and to evaluate the energy generated at each activation. In addition, experiments were conducted to investigate the effects of short use on the performance of the module.

The data obtained also contributed to a better understanding of the QST-MEH modules. Furthermore, this study aims to adapt the model described in [40] specifically for different activation speeds and to validate it with the experimental data.

Commercially available QST MEHs, such as the ECO 200, were originally designed for manual switching, which is characterized by low forces and speeds of almost zero. However, the pneumatic environment, which is characterized by much higher forces and speeds, offers significant untapped potential for energy recovery. The final part of the article will, therefore, show how the validated model can be used to optimize the design of an MEH specifically developed for the application at hand as a sensor inside a pneumatic cylinder. A genetic algorithm is used in conjunction with the model to determine the optimal MEH parameters for two objectives: maximizing energy harvesting and enhancing the correlation between the activation speed and output signals for better velocity detection.

The article introduces significant innovations compared to the state of the art in energy-harvesting systems, both in general and within the pneumatic domain. In general, it demonstrates the adaptation of QST-MEH devices to high-speed and high-force contexts like pneumatic systems.

Specifically for pneumatic systems, the study integrates a QST-MEH device into the cap of a pneumatic cylinder to recover energy from piston impacts. This solution enables the detection of both end-stroke and velocity, enhancing functionality while reducing the number of system components. Additionally, a Simulink model was developed and optimized to maximize energy recovery and enhance velocity detection capabilities. The study demonstrates that the performance can be optimized through improved design, significantly increasing energy efficiency and industrial applicability.

These contributions advance the understanding and practical application of energy-harvesting technologies in pneumatic systems, with potential implications for sustainability and self-powered industrial sensing solutions.

The article is structured as follows: After the description of the ECO 200 module, Section 2 describes the experimental setup, the tests performed, and the model. Section 3 is devoted to the results of the experiments in an open circuit and with a resistive load. Section 4 discusses the results of the experiments with regard to the effectiveness of speed detection, model validation, and device optimization. Conclusions are drawn in Section 5.

## 2. Materials and Methods

The beginning of this chapter is dedicated to a description of the ECO 200 module, which is the focus of this study. The following is a detailed description of the experimental setup and the methods used to measure the signals generated by the MEH when activated by a pneumatic cylinder at different speeds. In particular, voltage measurements were performed in two different cases: a no-load condition and with a resistance-load of 330 Ω. The analysis included important signal characteristics, including the peak voltage, time integral of the squared voltage, signal energy, rise time, and pulse width. Tests were also conducted to investigate the effects of short-term utilization. Finally, this chapter presents the model used to simulate the operation of an MEH and the methods used to validate it.

### 2.1. ECO 200 Energy Harvester

**Figure 1:**
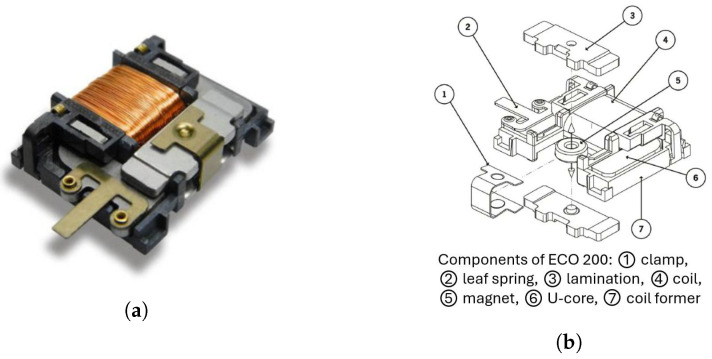
(**a**) Picture of the ECO 200 module; (**b**) Schematic representation of the ECO 200 components. Source: EnOcean GmbH datasheet.

The ECO 200, developed by EnOcean (Figure 1a), is a quasi-static mechanical energy harvester (QST-MEH) designed to convert mechanical motion into electrical energy through electromagnetic induction. The key elements of a QST-MEH are a permanent magnet, a soft-magnetic element and an electrical coil. Figure 1b shows the ECO200 main components.

**Figure 2:**
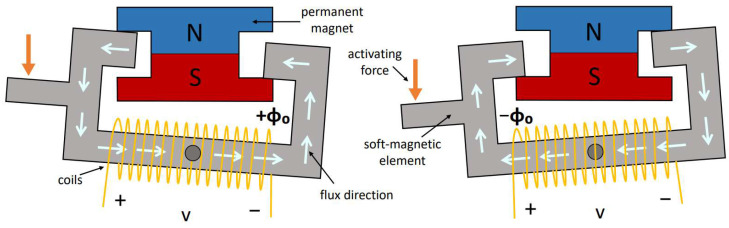
Schematic representation of the ECO 200 instant-swapping principle scheme.

The electrical coil surrounds a part of the soft-magnetic element, which is arranged with the permanent magnet to form a magnetic circuit. At least one of the soft-magnetic elements and the permanent magnet are mounted for rotary movement about an axis with respect to the other. A schematic view of the ECO 200 instant-swapping principle is shown in Figure 2. Within this configuration, there exist two stable states, upheld by the magnetic force, each associated with a magnetic flux passing through the soft element, equating to $\phi_{0}$ and $-\phi_{0}$. When an external activating force applied to the rotating component exceeds the magnetic holding force, the magnetic circuit instantly swaps between the configurations, resulting in a change in magnetic flux passing through the electrical coil equal to $2\phi_{0}$. Following Faraday’s law of induction, this transition induces a voltage pulse in the coil, effectively generating electrical energy. In ECO 200, the permanent magnetic element is made with a cylindrical permanent magnet with connected two iron lamination, and the rotating element is the soft-magnetic element (U-core).

**Figure 3:**
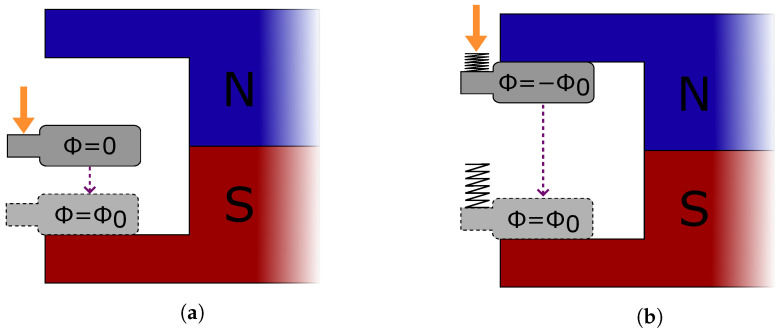
Comparison of MEHs without (**a**) and with (**b**) a pre-charging spring. The orange arrow represents the activation force. The instants represented are the ones right before and after the instant swapping to the second stable position when the device is activated with a quasi-static movement.

A distinctive feature of the ECO 200 is its spring-assisted pre-loading mechanism. A leaf spring serves as a force-applying medium, enabling instantaneous pole switching upon activation. This mechanism enhances electrical output for slow, singular activations. Without the spring, at negligible velocities, the magnetic transition would occur only when the attraction to the second position exceeds the first, resulting in a flux change from ϕ=0 to $\pm \phi_{0}$ (Figure 3a). However, with the spring, once the applied force surpasses the magnetic latching force, the released potential energy triggers an immediate $-\phi_{0}$ to $\phi_{0}$ transition, increasing energy generation (Figure 3b).

An additional component that must be used to develop the self-powered end-stroke sensor is the PTM 535 module. The PTM 535 is a wireless transmitter module by EnOcean designed to work with the ECO 200. It facilitates energy-harvesting applications like wireless switches and push buttons for EnOcean systems. The module operates at 868.3 MHz, uses ASK modulation, and supports a data rate of 125 kbps. It is capable of transmitting secure EnOcean radio telegrams with AES128 encryption. Compact and robust, the PTM 535 functions within a temperature range of −25 °C to 65 °C. Its design integrates features for high-security communication and configuration flexibility through its interfaces.

### 2.2. Experimental Setup Description

**Figure 4:**
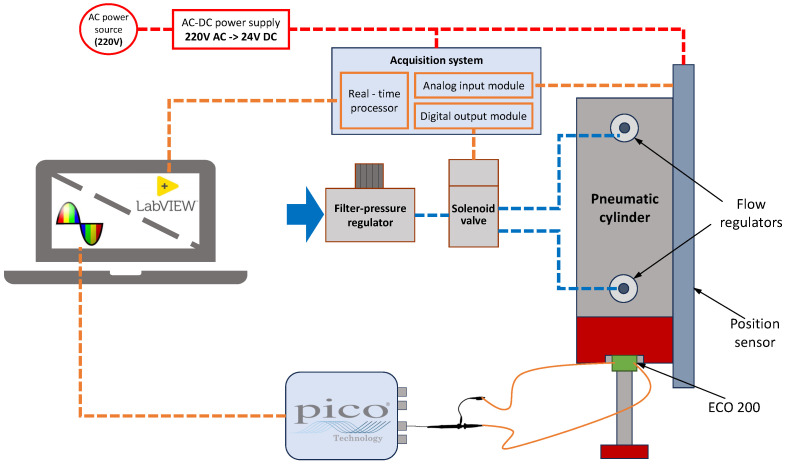
Experimental setup: electrical connections with sensors data and control commands in orange; electric power sources in red; pneumatic connections in blue.

The setup shown schematically in Figure 4 was chosen to carry out the experiments with the ECO 200 MEH integrated into a double-acting cylinder.

**Figure 5:**
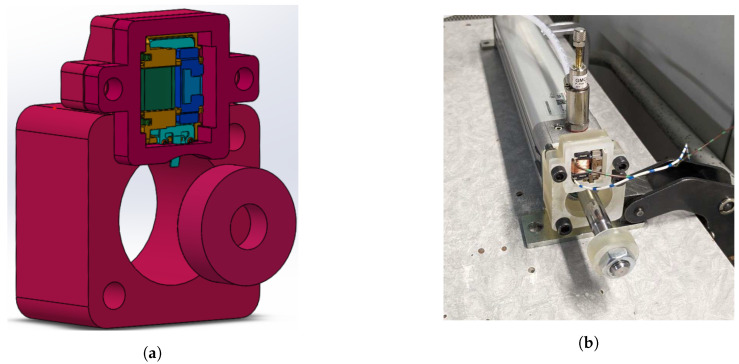
Support and mounting of the ECO 200 module on the pneumatic cylinder. (**a**) SolidWorks view of the support and activator. (**b**) Picture of the MEH mounted.

During the tests, the module was mounted on the front-end cap of the cylinder, and its switch was actuated at the end of the return stroke by an activator attached to the piston rod, as shown in Figure 5.

A PicoScope was used to record and measure the generated signal. A magnetic position sensor was used to track the position of the piston and determine the velocity. The sensor was positioned along the cylinder barrel to measure the position of the magnet integrated into the piston.

The pneumatic system used to activate the MEH comprised essential components such as the cylinder itself, a filter pressure regulator, a solenoid valve, and flow regulators.

The experimental setup employs an embedded real-time data acquisition system powered by a 24 V DC power supply, which converts 220 V AC mains voltage into a stable DC output. This power source also supplies the position sensor. The system comprises a chassis with integrated processing capabilities and modular input/output (I/O) functionalities. The board houses a real-time processor that executes the LabVIEW program developed to manage all experimental procedures. The chassis internally distributes power to its components and connects the I/O modules. These modules are responsible for signal management, including controlling the solenoid valve and acquiring data from the position sensor.

During all experiments, the pressure was set to 6 bar and the room temperature was kept constant at 23 °C.

The test-bed components are listed in Table 1.

During the tests, the energy-harvesting module was located in a special 3D-printed holder (Figure 5), which was attached to the front cap of the pneumatic cylinder. A special activator, also 3D-printed, was attached to the piston rod with a nut and locknut. Its function was to transfer force to the ECO 200 module’s switch when the piston reached the end of its stroke.

Care must be taken when positioning the activator as it determines the activation stroke of the energy-harvesting device, which influences the output voltage generated. With a too-long stroke, there is a risk of damaging the module. Thanks to the tests, the optimum position was found by moving the nut facing the front head 2.5 mm (two turns) away from the impact position.

The tests were carried out at different cylinder speeds. Speeds from 100 mm/s to 900 mm/s were tested, with multiples of 100 mm/s. Each speed was calculated from the position values as the average speed of the piston between the positions corresponding to 50 mm and 150 mm with respect to the inner position (with a total stroke of 200 mm). For each velocity value, a set of 30 trials was performed in which both the average velocity and the output voltage signal were recorded. During the trials, the speed did not correspond exactly to the desired value, but showed fluctuations around this value. However, it was found that there was no significant effect on the output waveforms within a range of ±10 mm/s from the desired value. Therefore, for each target activation speed, the mean value over the 30 trials was calculated for each signal feature, along with the standard deviation representing the degree of uncertainty. The analyzed signal features are as follows:

- Peak voltage: the maximum amplitude of the signal.
- Integral of the square of the voltage with respect to time, calculated over the duration of the signal pulse:
  (1) $$I=\int_{\mathrm{start}}^{\mathrm{end}}V{\left(t\right)}^{2}\;dt$$
- Total energy of the signal: evaluated when a resistance *R* is connected, using the time integral of the squared voltage:
  (2) $$E=\frac{I}{R}$$
- Rise time of the pulse: the time taken for the signal to transition from 10% to 90% of its maximum value.
- Pulse width: the time interval between the instants where the signal reaches 50% of its maximum value.

To ensure a comprehensive evaluation of the energy harvester’s performance, voltage signal testing was conducted under two distinct conditions: no-load and loaded. The loaded condition is fundamental for the intended application. Conversely, no-load testing provides critical insights into the intrinsic properties of the device, independent of any external load. By isolating the energy harvester from external influences, this testing condition facilitates a deeper understanding of its baseline performance characteristics, such as the peak voltage and the shape of the generated waveforms. This knowledge is essential for optimizing the device’s design and identifying potential improvements in energy conversion efficiency.

Together, these testing conditions offer a holistic understanding of the energy harvester’s capabilities, bridging the gap between theoretical performance and practical application.

Three types of test were carried out in the order indicated:

- First no-load test: to test the harvester’s internal voltage capability when it is not supplying power to an external device.
- Load test with a resistor of 330 Ω: with a resistive load, it is possible to analyze the current and energy values.
- Second no-load test: Comparison with the first no-load test results made it possible to observe the effects of short-term use on the energy harvester.

### 2.3. Model of the System

**Figure 6:**
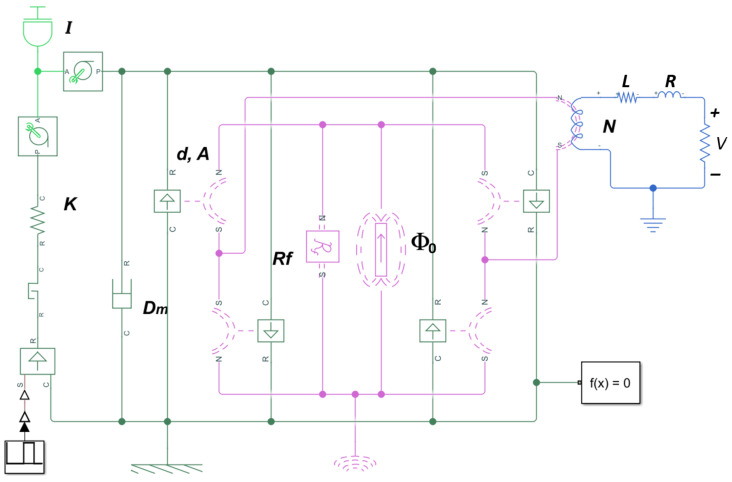
Simulink model of a QST-MEH; mechanical connections are depicted in green, magnetic connections in fuchsia, and electrical connections in blue.

Figure 6 shows the model developed in the Matlab Simulink environment, used to simulate the signal waveform generated by a QST-MEH, in particular, the ECO 200, when activated at different speeds. To capture the transient response after switching a QST-MEH, the complex coupled dynamics between mechanical, magnetic, and electrical domains must be considered. The construction of an analytical model with closed-form expressions proves to be difficult; therefore, the Simulink model shown in Figure 6 provides a simple but effective numerical simulation approach [40].

The model parameters that represent a specific QST-MEH include the elasticity constant of the spring acting as a switch *K*, the stroke of the moving part when activated *d*, the cross-sectional area of the magnetic circuit *A*, the magnetic flux generated by the permanent magnet $\Phi_{0}$, the inertia (or mass) of the moving part *I*, and the number of coils *N* in the solenoid coil. Other parameters, such as the mechanical damping *Dm* or the reluctance associated with the leakage flux *Rf*, the inductance *L*, and the internal resistance of the module *R*, are assumed to be constants or a function of the other parameters. The parameters used for the ECO 200 are listed in Table 2 and all simulations were performed under the condition of a 330 Ω load.

The Simulink model was subjected to two different verification methods. First, the outputs of the model were compared with the averaged experimental waveforms obtained from the load test. Then, the analysis was extended to all analyzed signal features to allow a comprehensive evaluation of the model performance.

Subsequently, an evaluation of the ECO-200 parameters was performed using genetic algorithms. This estimation consisted of fine-tuning the model parameters to replicate the measured waveforms as closely as possible. The estimated parameters were then compared to the actual parameters to further evaluate the accuracy of the model.

## 3. Results

### 3.1. No-Load Tests

This section presents the experimental results of tests carried out with the ECO 200 module under no-load conditions.

**Figure 7:**
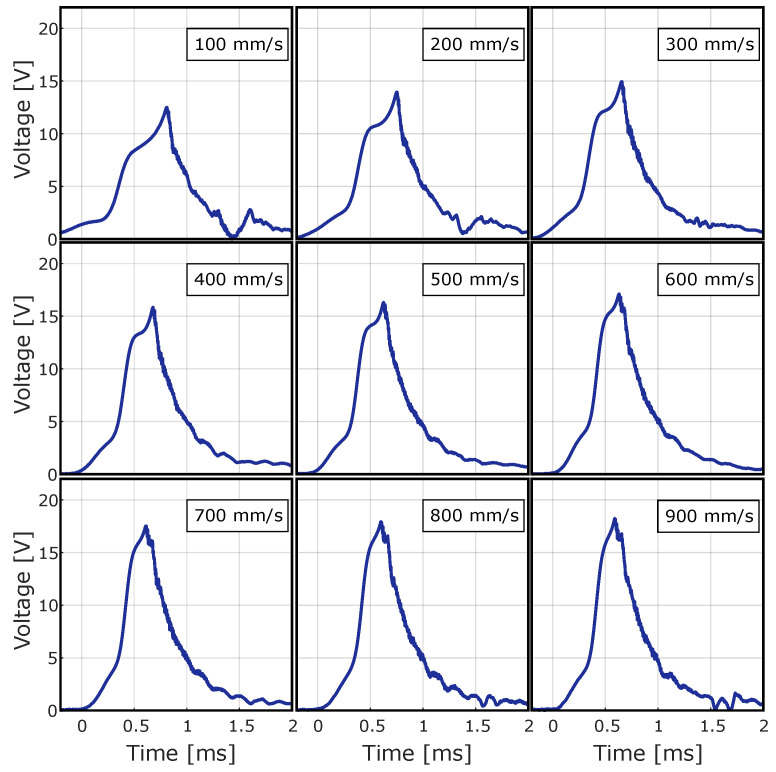
Average waveforms produced by the QST-MEH ECO 200 module under open-circuit conditions during the first no-load test.

Figure 7 shows the average waveforms recorded during the tests at all activation speeds considered.

These signals exhibit a consistent rise and fall pattern and show a short-term peak that is usually shorter than 2 milliseconds. The effect of the activation speed becomes clear as the intensity of the signals increases with higher amplitudes and accelerated pulse rates.

**Figure 8:**
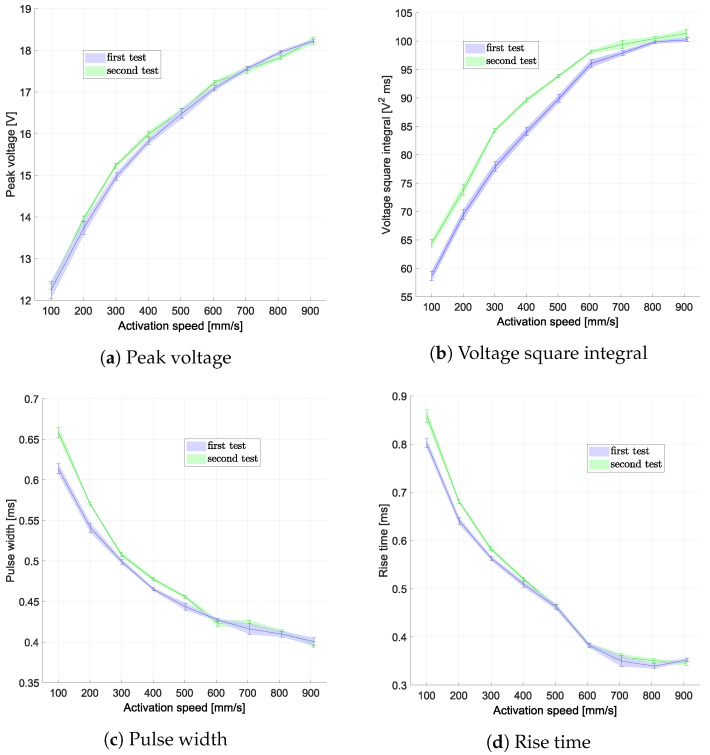
Features evaluated from signals related to different activation speeds in the first and second no-load tests.

The results presented in Figure 8 illustrate the relationship between the activation speed and key signal characteristics, including the peak voltage, voltage square integral, pulse width, and rise time, obtained during the no-load tests. A clear trend is evident across all analyzed features, demonstrating the influence of the activation speed on the harvester’s output.

Specifically, the peak voltage and voltage square integral show a pronounced increase with rising activation speed, following second-order polynomial behavior. This trend highlights the enhanced energy generation capability of the device at higher speeds, which is consistent with the principles of electromagnetic induction. Conversely, both the pulse width and rise time decrease as the activation speed increases, reflecting the shorter duration and faster dynamics of the generated signals at higher velocities. These findings confirm the strong correlation between the activation speed and the harvester’s signal characteristics. The trends observed not only validate the feasibility of using the signal for velocity sensing but also provide valuable data for optimizing the device’s design.

The second test, carried out without load, was used to analyze any changes in the behavior of the MEH after a short period of use. In the time between the first and second no-load tests, the module was activated approximately 600 times (30 times for 9 speeds, first without and then with load, plus additional activations for speed calibration). There were almost no differences in the peak voltages, while the other parameters showed significant differences, especially the voltage square integral, with a relative error of up to 10% at the lower speeds. A comprehensive set of all results with uncertainty values and relative differences between the tests is shown in Table 3.

### 3.2. Load Test and Simulations

In this section, the results of the experiments with a 330 Ω resistor are presented and compared with the predictions of the model to assess its validity. These results are of particular importance as the inclusion of a resistor allows the evaluation of the current amplitude and the total energy of the signal.

**Figure 9:**
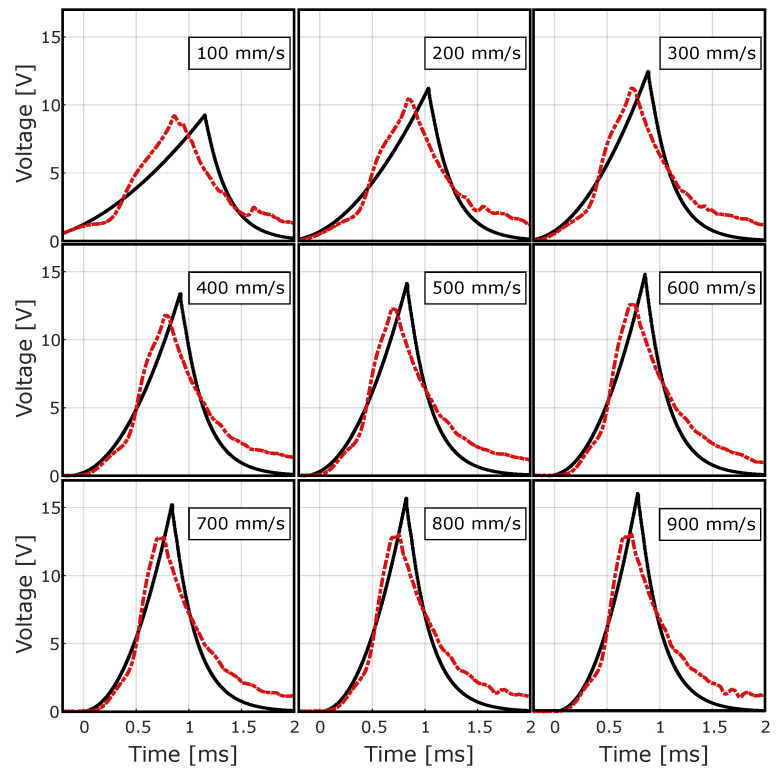
Simulated output voltage signals (solid black line), juxtaposed to average signals of load test (red dashed line).

Figure 9 shows the Simulink model outputs of ECO 200 at the specified activation speeds, which are compared with the experimental measurements.

**Figure 10:**
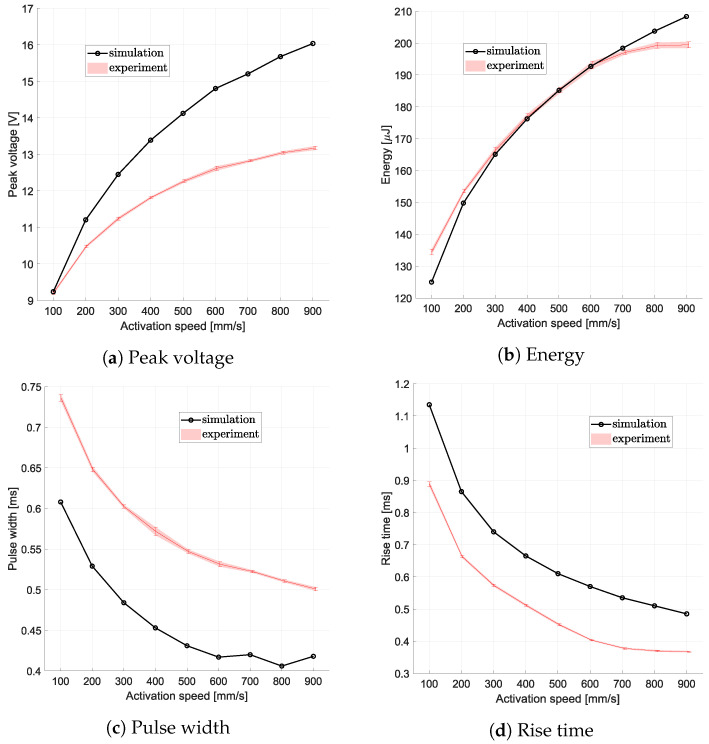
Features trends versus activation speed for simulation and load tests.

Experimental and simulated waveforms have comparable shapes, although the simulated signals have more pronounced peaks. Figure 10 shows a detailed comparison of the evaluated signal properties at different activation speeds.

The peak voltage values at 100 mm/s match exactly, but deviate significantly from the experimental data at higher speeds. The signal energy shows almost complete agreement between the experimental and simulated results. The pulse width of the simulated signals at the different speeds has a similar trend to the experimental signals, but shows a negative offset. The trends of the experimental and simulated rise time at different velocities are also very similar, but there is an offset, in this case, a positive one.

Table 4 gives a comprehensive overview of the results, including the relative errors between the simulations and the experimental data.

To further validate the model, the most important parameters characterizing the ECO 200 module were estimated from the experiments using genetic algorithms. Table 5 reports the estimated parameters together with the actual parameters and provides the relative error values. Despite an error range of up to 30%, the estimated values are of the same order of magnitude as the actual parameters, indicating a reasonable approximation.

## 4. Discussion

### 4.1. Interpretation of the Experimental Outcomes

The results of the experimental tests consistently showed a significant correlation between the waveforms generated by the ECO 200 module and its activation speed. In particular, higher impact velocities corresponded to more intense signals, resulting in more pronounced voltage peaks, higher energy output, and faster pulses with shorter rise times and pulse widths.

This behavior can be traced back to the basic operating principle of the QST-MEH devices, which work based on electromagnetic induction. The activation switch is connected to a moving component that dynamically changes the magnetic flux flowing through the coils, inducing a voltage according to Faraday’s law (Equation (3)). Consequently, faster activations lead to faster changes in the magnetic flux and thus to higher induced voltages. It is important to clarify that the speed of the moving component mentioned here is not the same as the activation speed. Rather, it is the result of complex mechanical, electrical, and magnetic interactions within the elements of the MEH.

(3) $$V=-\frac{d\Phi }{dt}$$

### 4.2. Self-Powered End-Stroke Sensor Feasibility

As expected, the experimental results show that the energy generated by the sensor during impact, even at the lowest speed, is sufficient to produce an end-stroke signal. To this end, the ECO 200 module was integrated with the PTM 535 module, which rectifies and transmits the signal containing the end-stroke information. This system proves to be effective as a self-powered end-stroke sensor.

### 4.3. Self-Powered Speed Sensor Feasibility

The observed trends in the analyzed traits showed an overall polynomial pattern, with higher slopes at lower speeds and only marginal differences at higher speeds. The range of impact velocities investigated, from 100 mm/s to 900 mm/s, corresponds to typical applications for pneumatic actuators. For a possible application as a stand-alone velocity sensor, the ECO 200 device demonstrates the ability to generate recognizable signals that allow differentiation of velocity in the range of 100 mm/s to 600 mm/s. However, problems can occur when differentiating speeds between 600 mm/s and 900 mm/s.

Another important point for the feasibility of a self-powered speed sensor is the energy generated by the MEH. The generated signals must have sufficient energy to fulfill three critical phases:

- Power a device, possibly a specialized circuitry or a microprocessor, responsible for converting certain signal attributes into activation speed information.
- Maintain the essential waveform characteristics required for speed detection during the process.
- Power a radio transmitter that broadcasts this derived information.

In this context, the aim of the study was to provide basic data on the energy yield in order to develop a suitable solution. The experiments showed that the energy output has a direct correlation with the activation speed and lies between 130 μJ at 100 mm/s and 210 μJ at 900 mm/s.

### 4.4. Brief Usage Effects

A comparative analysis of the experimental results of the first and second tests without load showed that the short-term use of the module (approx. 600 activations) had a noticeable effect. The differences between these tests manifested themselves more clearly in larger pulse widths and higher total voltage square integral values. Remarkably, these differences lacked consistency across all activation speeds, exhibiting varying offsets.

This discrepancy is likely the result of mechanical changes within the device due to the much higher kinetic energy associated with the piston strokes, as opposed to the manual switching for which the device is primarily designed. There are parameters such as the elasticity constant of the toggle switch or the total stroke of the moving part during its activation that are likely to be altered by strong excitation.

EnOcean advises against deploying the module in mechanically challenging environments characterized by high shock [41].

Further research is essential to gain a comprehensive understanding of the underlying causes of the observed fluctuations. Such an analysis would not only help to develop effective strategies to cope with these changes, but would also allow the investigation of possible effects after prolonged and continuous use of the module.

### 4.5. Model Validation

The simulation results provide very good results and successfully reproduced waveforms that are very similar to the experimental data at all activation speeds.

In particular, the energy output is almost identical to the experimental results. This result is of great importance as the model is primarily intended to serve as a tool for the development of new energy harvesters optimized for improved energy harvesting in pneumatic environments.

Despite this alignment, alterations in waveforms shapes lead to discrepancies between the experimental and simulated signals on rise time and pulse width. In the worst-case scenario, the model overestimates the rise time by approximately 27% and underestimates the pulse width by about 20%. However, it is important to emphasize that despite these deviations both features still show a significant correlation with the impact velocity, a behavior consistent with the experimental results.

Furthermore, the results for the peak are not fully representative of the actual values and deviate from the actual trends. The deviation increases with the activation speed, and, in the worst case, the peak amplitude is overestimated by 23%. However, they do provide some insight into the signal intensity. For a more accurate interpretation, it is advisable to focus on the overall signal shape and energy when using the model, rather than relying only on the peak values.

In addition, the parameter identification through genetic algorithms confirmed the reliability of the model. The objective function was adjusted to emphasize the evaluation of the signal integral and provide parameters that confirm the accuracy of the model and, most importantly, show agreement with the actual experimental results.

### 4.6. Design of an Optimized Energy Harvester

The validated model provides a tool for the development of a new MEH specifically designed for pneumatic environments. Pneumatic cylinders operate at higher speeds and exert a higher force, resulting in greater kinetic energy than the manual switching for which the QST MEHs, such as ECO 200, were originally designed. In addition, the ECO 200 module was designed primarily for actuation at lower speeds, and the higher tensions generated by the higher actuation speeds are incidental results resulting from its operating principle. At lower speeds, most of the energy converted to electrical energy comes from the potential energy stored in the spring mechanism. However, a more appropriate design approach would shift the primary energy generation from potential energy to kinetic energy, which would better meet the requirements of pneumatic cylinders.

In designing this new MEH, we focused on the total signal energy parameter, as the model has demonstrated high reliability in reproducing this parameter across all activation velocities. This approach serves two purposes: it enables the optimization of energy harvesting, which would be essential for powering future circuits designed to detect and transmit the arrival velocity, while also allowing the energy level itself to be used as a parameter for distinguishing different velocities.

#### 4.6.1. Application of Genetic Algorithms

An illustrative case study is presented in which the model was used in conjunction with genetic algorithms to evaluate the optimal parameters of an MEH for energy harvesting and signal differentiation in the context of pneumatic systems.

The optimization problem for the studied application is characterized by many local minima. When there are many local minima in an optimization problem, the most effective algorithms tend to be those that can explore large regions of the search space and escape local minima to find more global solutions. Genetic algorithms, which are inspired by natural evolutionary processes, are particularly suited for problems with many local minima. They use mechanisms such as selection, mutation, and crossover to explore the search space broadly, increasing the chances of finding globally optimal solutions while avoiding getting trapped in local minima.

The pneumatic cylinder considered in this study was the same as the one used in the experiments and the objective function chosen is the following:

(4) $$J_{out}=-\sum_{i=1}^{9}E_{i}-\alpha \sum_{i=2}^{9}\frac{\left(E_{i}-E_{i-1}\right)}{E_{i}}.$$

In this equation, $E_{i}$ is the energy measured at activation speed *i*, with speeds ranging from 100 mm/s to 900 mm/s, and α is a constant used to weight the two parts of the equation. The first term, the sum of the energy values across all the activation speeds, corresponds to the desired improvements in energy extraction. The second term aims to enhance the effects of the activation speed on the generated signals.

Constraints were applied considering the maximum activation force required, assuming a conservative scenario where the pneumatic cylinder was activated under a pressure of 1 bar and without any load. Additional restrictions were placed on the overall dimensions of the device to ensure that it would fit into the end cap of the cylinder.

#### 4.6.2. Optimization Results

The optimization process led to the determination of the parameters shown in Table 6.

**Figure 11:**
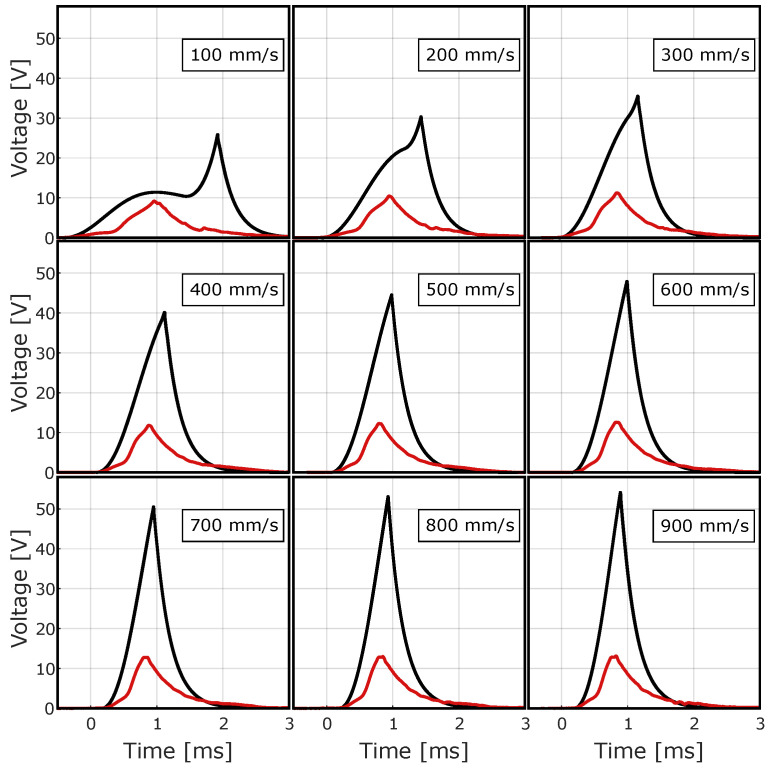
Simulated waveforms outputs of the optimized MEH (black line) juxtaposed to average waveforms of the load tests (red line).

The simulated signals generated by the resulting energy harvester with a load of 330 Ω (Figure 11) have a significantly higher amplitude compared to the signals generated by the ECO 200.

**Figure 12:**
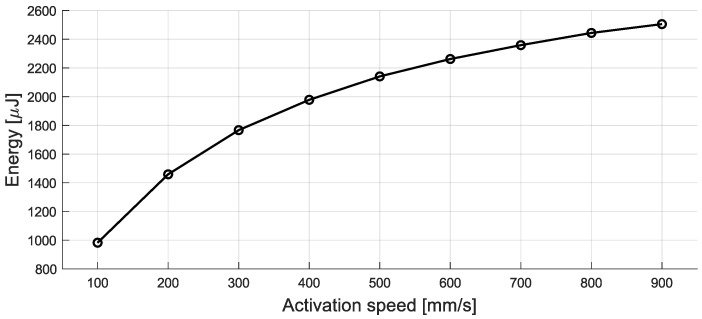
Simulated energy trend with respect to activation speed of the optimized MEH.

As a direct consequence, the extracted energy is much higher—about ten times higher—and ranges between 1 mJ and 2.5 mJ (Figure 12).

The influence of the velocity on the overall shapes of the signals and the resulting amounts of energy is obviously more pronounced, although differentiation in the velocity range from 800 mm/s to 900 mm/s could be a challenge. Among the most important parameters contributing to these improved performances is a higher magnetic flux $\Phi_{0}$. This leads to increased flux change rates and, therefore, higher voltage values, which, in turn, require a stronger activation force. Equally important is the increased elastic constant *K* of the switch. A more stable switch transfers kinetic energy directly to the device, rather than accumulating potential energy via a spring mechanism, and thus benefits from the relatively high speeds. Further contributions result from the increased number of coils, among other parameters.

## 5. Conclusions and Recommendations for Future Work

In this article, a novel application of mechanical energy harvesters as self-powered end-stroke and speed sensors for pneumatic systems is analyzed. In particular, the proposed approach consists of integrating a commercial module that converts part of the excess energy of the piston impact into an electrical signal. This signal is used to determine the impact velocity and transmit relevant information such as the final stroke. The investigation began with the experimental analysis of an energy-harvesting device, specifically, EnOcean’s ECO 200, and extended to its modeling and optimization through genetic algorithms for improved energy harvesting.

The experiments served as a preliminary study for the feasibility of the solution, with the following results, in particular:

- The experiments showed a clear correlation between the characteristics of the waveforms generated by ECO 200 and the activation speed with a pneumatic cylinder. Faster impacts resulted in more pronounced signals, i.e., a higher voltage peak, higher energy output, and faster pulse rates. It is therefore, possible to recognize the impact speed of the piston from the electrical signal of the energy harvester.
- The total amount of energy generated by the module was revealed and provides crucial information for the development and deployment of the planned solution.
- The effects of a short-term use of the device on the signals were measured and showed a moderate but not negligible influence on the generated waveforms.
- The ECO 200 module, designed to function as a self-powered switch, is activated when the piston impacts the end cap. The study demonstrated that the generated energy, rectified by the PTM 535 radio module (also by EnOcean), can be used to transmit a Bluetooth signal indicating the reach of the end-stroke.

The data collected during the measurements served the second purpose of validating a model of the energy harvester. The following results were obtained:

- Despite noticeable errors in certain signal characteristics, the basic waveform and, more importantly, the overall energy levels, match the real results exactly. The agreement justifies the usefulness of the model for the development of innovative energy-harvesting devices.
- By optimizing the primary model parameters using genetic algorithms, it was possible to design a new energy harvester. According to the simulations, it would harvest ten times more energy than the ECO 200 while generating signals with higher differentiation.

The positive results showed the possibility of implementing the solution in several aspects, but further studies should be carried out in order to:

- Implement a solution with a microprocessor or a special circuit to detect the impact velocity from the signal of the energy harvester and transmit the value to a receiver. Two potential approaches could be explored:
  1. Developing a circuit to rectify the signal into a DC voltage with an amplitude proportional to the signal’s energy. This voltage level would then be used to generate radio signals at different frequencies, effectively encoding the impact velocity information through frequency modulation.
  2. Alternatively, the circuit could rectify the signal into a DC voltage of fixed amplitude, but with a duration proportional to the signal’s energy. This varying duration would then be used to generate radio signals with constant characteristics but different lengths, where the transmission duration would directly correspond to the impact velocity.
- Future work will focus on extended operational testing and reliability analysis under real-world conditions. These studies will evaluate potential degradation in the device’s performance due to mechanical wear, environmental factors, and repeated use over time. The insights gained from such investigations will help identify key factors affecting the long-term stability and enable the development of design enhancements to mitigate these issues. This approach will not only validate the feasibility of the system for sustained industrial use but also provide critical data for optimizing the durability and robustness of energy harvesters in pneumatic applications. By incorporating long-term stability considerations, the system can be further aligned with the practical requirements of self-powered sensing solutions in demanding environments.
- Design and develop a prototype energy harvester that incorporates the optimal device properties identified through the optimization process. The analysis conducted in this study, by determining the ideal parameters for energy harvesting, serves as the foundation for developing an optimized end-of-stroke sensor. Testing this prototype would validate the model’s predictions and mark a significant step toward realizing a fully functional self-powered sensor.

To summarize, this work highlights the potential of energy-harvesting technology in pneumatic environments. The fusion of experimental analysis, modeling, and optimization techniques has provided valuable insight into the integration of energy-harvesting devices in pneumatic cylinders. The hope is that this work can serve as a springboard for further research aimed at refined models, robust designs, and broader practical applications of MEH technology in various fields.

## Figures and Tables

**Table 1:** Instruments and components.

Instrument/Component	Model/Specification
Position Sensor	Gefran MK4-A-A-0500
Acquisition System	Hardware National Instruments
Oscilloscope	PicoScope
Energy-Harvesting Module	EnOcean ECO 200
Pneumatic Cylinder	Camozzi 61M2P032A0200 (damping gasket removed)
Flow Regulators n° 2	Camozzi GMCU 903-1/8-6
Solenoid Valve	Camozzi 354-015-02
Filter-Pressure Regulator	Camozzi MX2-1/2-FR0000
Anchors n° 2	Camozzi B-6E-320
Stem Nuts n° 2	Camozzi U-25-32
ECO 200 Support	3D Printed
ECO 200 Activator	3D Printed

**Table 2:** Parameters used for simulation for ECO 200.

Parameter	Value	Parameter	Value
$\Phi_{0}$	5.45 μWb	N	763
K	5400 N/m	Rf	$4.8\times 10^{8}$ 1/H
I	108 g ${\mathrm{mm}}^{2}$	Dm	$1\times 10^{-4}$ N s/m
d	0.4 mm	R	27.61 Ω
A	8.75 ${\mathrm{mm}}^{2}$	L	36.84 mH

**Table 3:** Mean values of the features at different activation speeds evaluated in the first and second no-load tests.

		Activation Speed [mm/s]								
		100	200	300	400	500	600	700	800	900
Peak voltage [V]	1st test	12.24±0.08	13.74±0.07	14.97±0.06	15.82±0.07	16.48±0.05	17.09±0.04	17.57±0.08	17.96±0.05	18.23±0.09
	2nd test	12.33±0.08	13.95±0.07	15.23±0.06	15.99±0.07	16.55±0.05	17.23±0.04	17.53±0.08	17.83±0.05	18.23±0.09
	Relative error	0.74%	1.53%	1.74%	1.07%	0.42%	0.82%	0.23%	0.72%	0.00%
Voltage square integral [$V^{2}$ms]	1st test	58.66±0.84	69.52±0.86	77.9±0.87	84.06±0.72	89.91±0.67	96.06±0.67	97.92±0.4	99.89±0.33	100.24±0.34
	2nd test	64.44±0.68	73.8±0.93	84.25±0.35	89.62±0.37	93.83±0.29	98.15±0.27	99.41±0.71	100.45±0.38	101.4±0.67
	Relative error	9.85%	6.16%	8.15%	6.61%	4.36%	2.18%	1.52%	0.56%	1.16%
Pulse width [μs]	1st test	614±6	541±6	499±3	465±2	444±4	427±2	416±7	410±4	401±5
	2nd test	658±6	570±1	508±3	478±2	456±2	423±4	423±4	413±2	397±4
	Relative error	7.17%	5.36%	1.80%	2.80%	2.70%	0.94%	1.68%	0.73%	1.00%
Rise time [μs]	1st test	801±9	640±7	562±4	509±6	462±6	382±5	350±11	339±5	352±4
	2nd test	858±13	681±4	583±4	520±3	463±5	384±3	359±6	351±4	346±5
	Relative error	7.12%	6.41%	3.74%	2.16%	0.22%	0.52%	2.57%	3.54%	1.70%

**Table 4:** Mean values of the features at different activation speeds evaluated in the load tests.

		Activation Speed [mm/s]								
		100	200	300	400	500	600	700	800	900
Peak voltage [V]	Test 330Ω	9.21±0.04	10.48±0.03	11.24±0.04	11.82±0.03	12.27±0.04	12.62±0.06	12.83±0.03	13.05±0.04	13.18±0.05
	Simulation	9.24	11.21	12.45	13.39	14.13	14.81	15.21	15.68	16.04
	Relative error	0.33%	6.97%	10.77%	13.28%	15.16%	17.35%	18.55%	20.15%	21.70%
Peak current [mA]	Test 330 Ω	27.90±0.11	31.77±0.10	34.06±0.13	35.82±0.09	37.19±0.12	38.24±0.18	38.87±0.10	39.55±0.12	39.93±0.16
	Simulation	28.00	33.97	37.73	40.58	42.82	44.88	46.09	47.52	48.61
	Relative error	0.36%	6.92%	10.78%	13.29%	15.14%	17.36%	18.57%	20.15%	21.74%
Energy [μJ]	Test 330 Ω	134.5±0.9	153.5±0.6	166.3±0.9	177±0.8	185.3±0.8	193.1±1.2	197.1±0.6	199.3±0.9	199.4±1
	Simulation	125.0	149.8	165.1	176.2	185.2	192.7	198.4	203.7	208.3
	Relative error	7.06%	2.41%	0.72%	0.45%	0.05%	0.21%	0.66%	2.21%	4.46%
Pulse width [μs]	Test 330 Ω	736±4	648±3	602±2	572±5	547±2	531±3	522±1	511±2	501±2
	Simulation	699	569	504	464	439	414	404	389	379
	Relative error	5.03%	12.19%	16.28%	18.88%	19.74%	22.03%	22.61%	23.87%	24.35%
Rise time [μs]	Test 330 Ω	888±8	663±4	574±4	511±3	451±3	403±2	377±2	370±3	368±2
	Simulation	1135	865	740	665	610	570	535	510	485
	Relative error	27.82%	30.47%	28.92%	30.14%	35.25%	41.44%	41.91%	37.84%	31.79%

**Table 5:** Comparison between the actual parameters of ECO 200 and the parameters estimated with genetic algorithms.

Parameter	Real Value	Estimated Value	Relative Error
K	5400 N/m	4200 N/m	22.22%
$\Phi_{0}$	5.46 μWb	5.34 μWb	2.23%
I	108 g ${\mathrm{mm}}^{2}$	95 g ${\mathrm{mm}}^{2}$	12.03%
d	0.4 mm	0.32 mm	20.00%
A	11 ${\mathrm{mm}}^{2}$	14.6 ${\mathrm{mm}}^{2}$	32.72%
N	763	853	11.80%

**Table 6:** Parameters of the optimized MEH.

Parameter	Value	Parameter	Value
K	20,000 N/m	d	0.8 mm
$\Phi_{0}$	12.6 μWb	A	11 ${\mathrm{mm}}^{2}$
I	108 g ${\mathrm{mm}}^{2}$	N	1140

## Data Availability

Data are contained within the article.
